# Supplementary material for: Cutin from Solanum Myriacanthum Dunal and Solanum Aculeatissimum Jacq. as a Potential Raw Material for Biopolymers
Source: Polymers (Basel). 2020 Aug 28;12(9):1945. doi: 10.3390/polym12091945 (PMC7565047; doi:10.3390/polym12091945)

SUPLEMENTARY MATERIAL

1H NMR of the 18-hydroxy-9S,10R-epoxy-octadecanoic acid

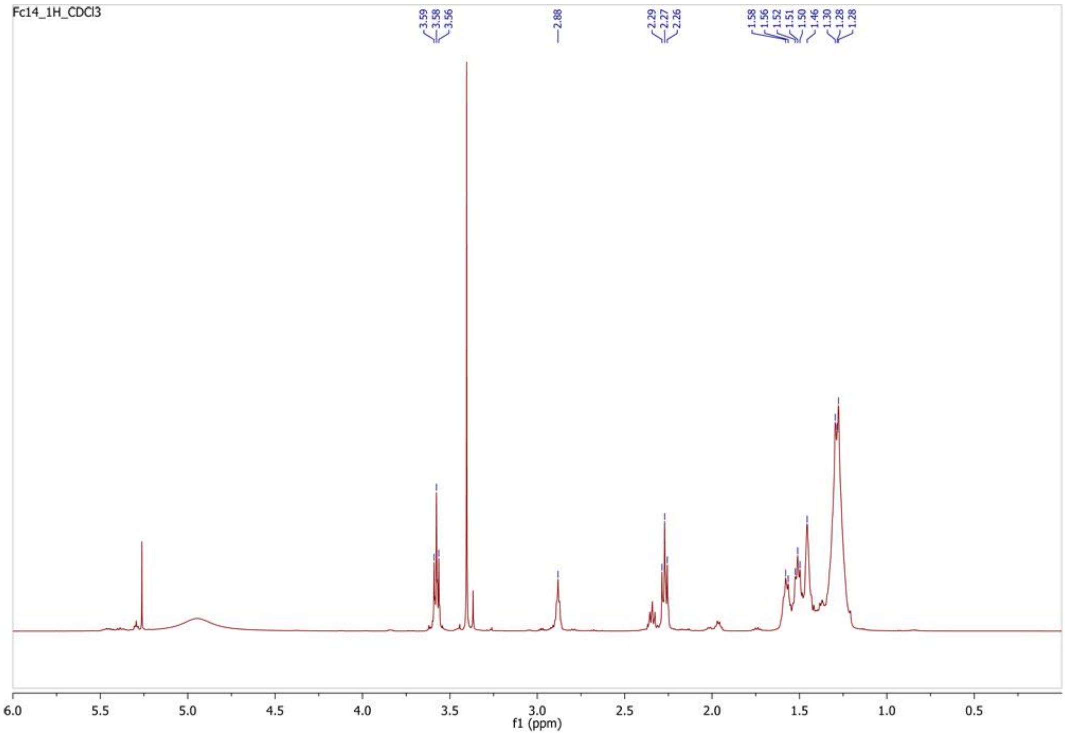

Comparative spectra of the simulated and experimental 1H NMR of the 18-hydroxy-9S,10R-epoxy-octadecanoic acid

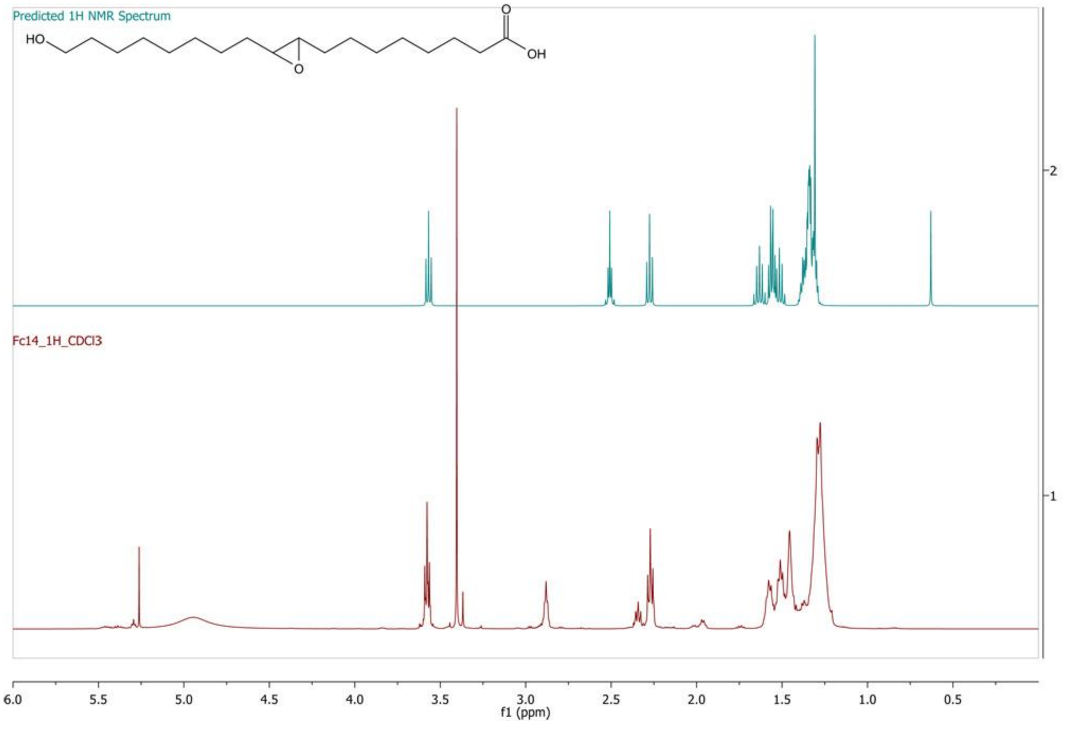

13C NMR of the 18-hydroxy-9S,10R-epoxy-octadecanoic acid

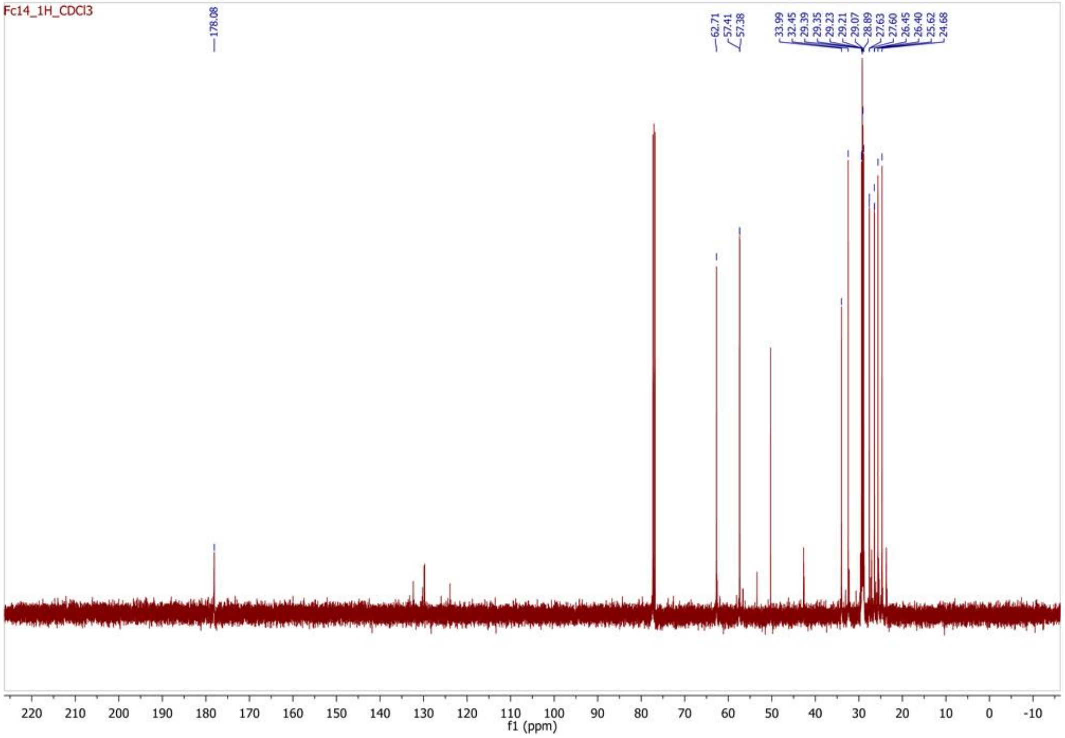

Comparative spectra of the simulated and experimental 13C NMR of the 18-hydroxy-9S,10R-epoxy-octadecanoic acid

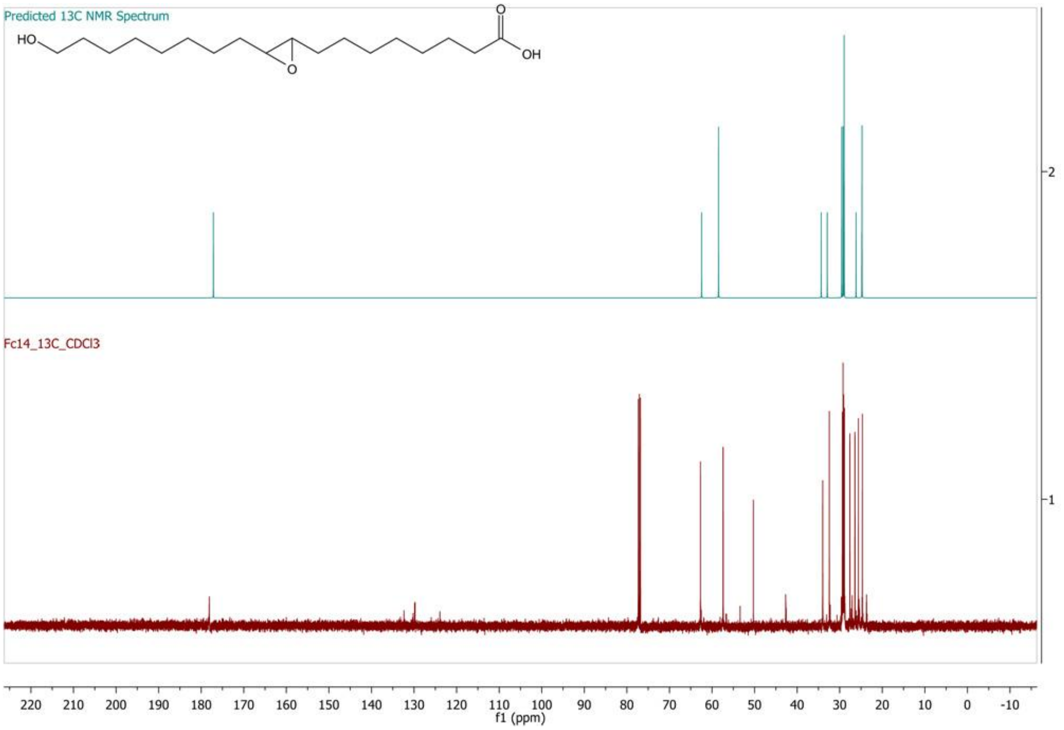

HMBC NMR of the 18-hydroxy-9S,10R-epoxy-octadecanoic acid

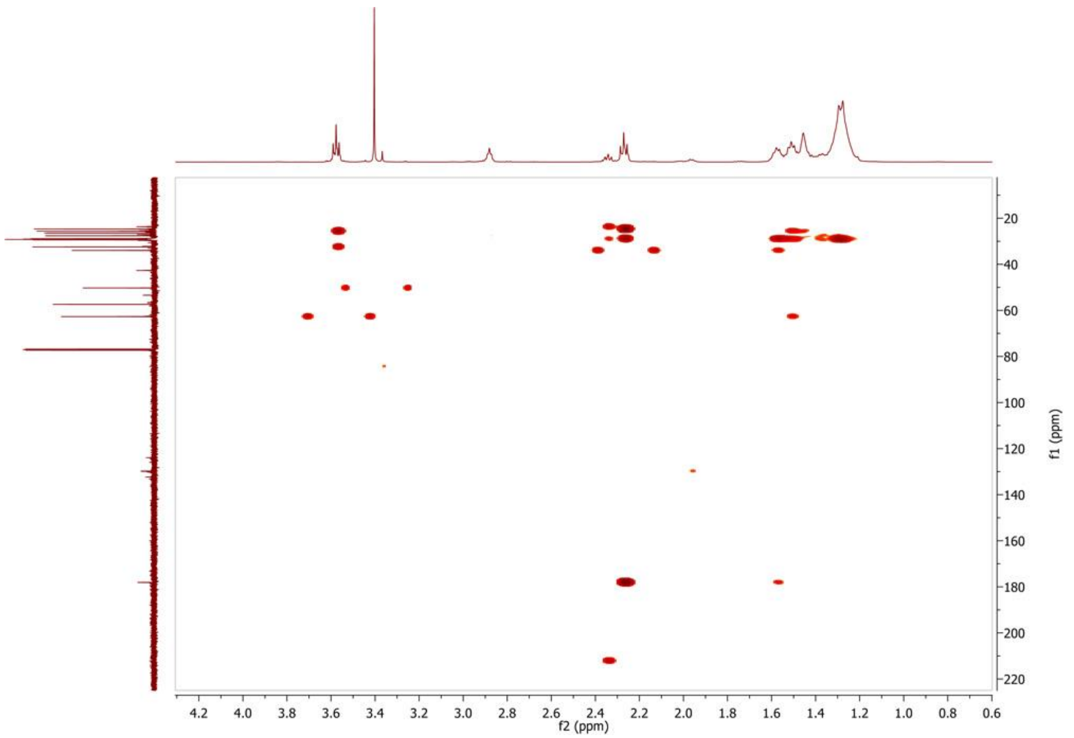

ESI spectra of the 18-hydroxy-9S,10R-epoxy-octadecanoic acid

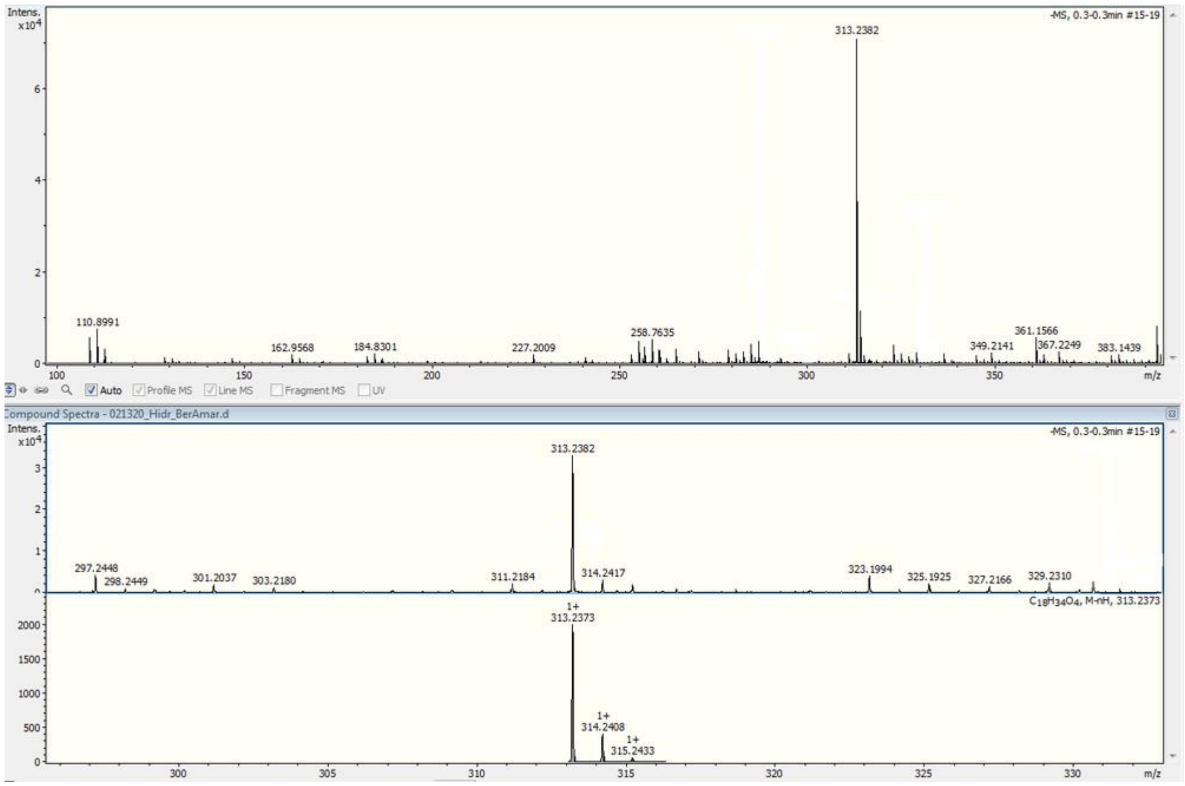

DIESI-MS spectra of the hydrolyzed *S. aculeatissimum* cutin

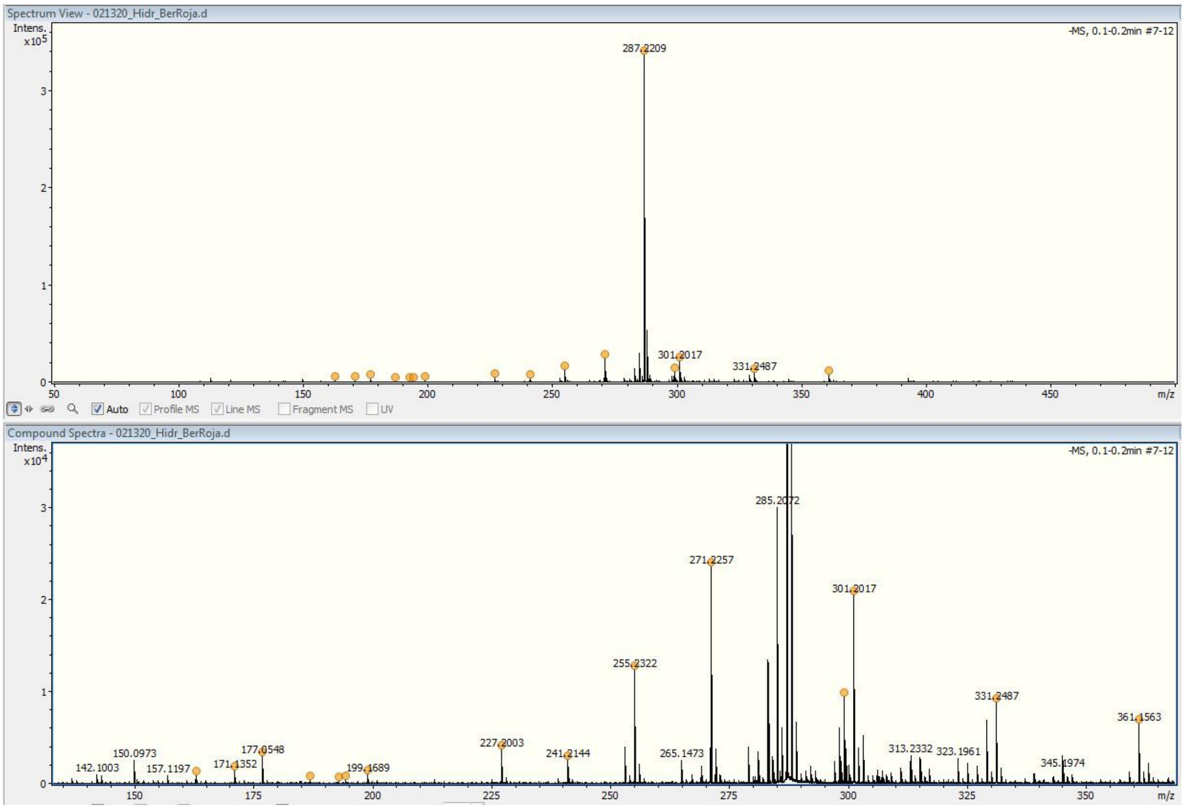

DIESI-MS spectra of the hydrolyzed *S. myriacanthum* cutin

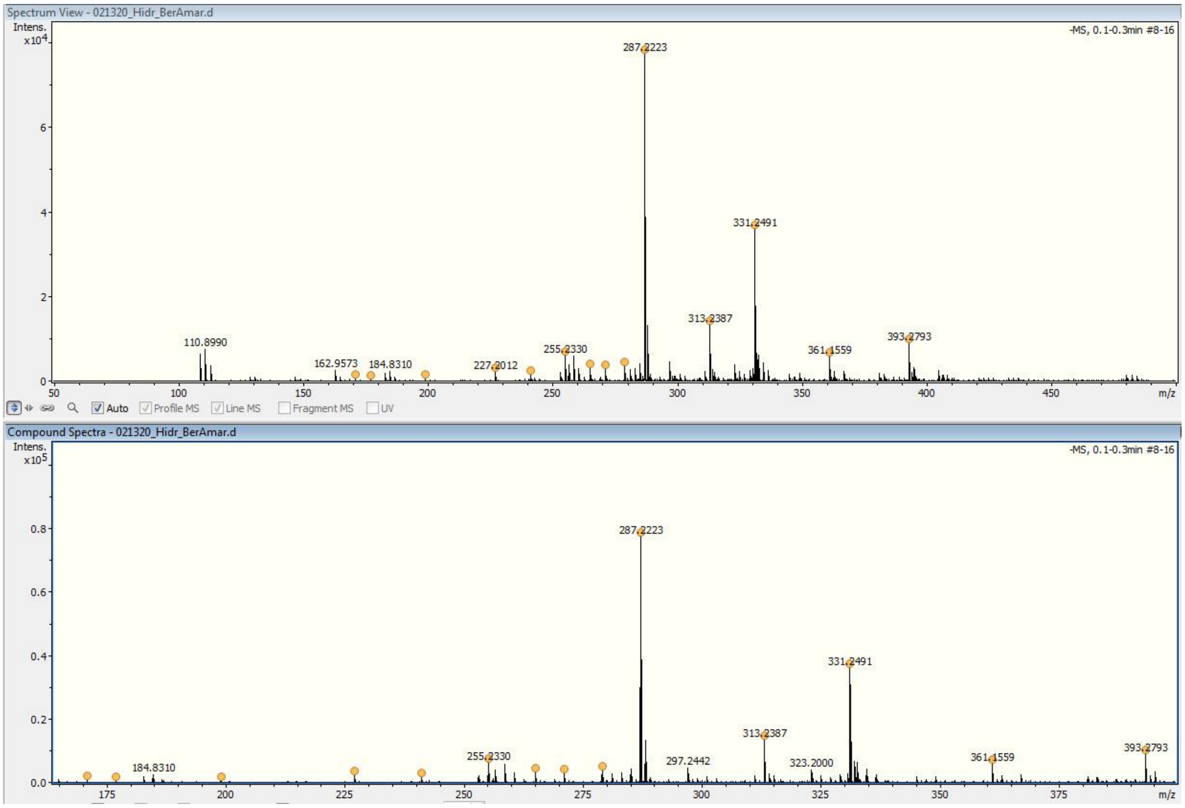

Supplement: Supplementary file 1 [file polymers-12-01945-s001.pdf]
